# Association of self-leadership and epidemic risk perception on quality of life in post-pandemic mainland of China: a cross-sectional study

**DOI:** 10.3389/fpubh.2024.1394416

**Published:** 2024-06-25

**Authors:** Jiaju Ren, Yanbo Zhu, Yuan He, Xinyuan Zhao, Guoming Pang, Liqun Long, Qian Zhang

**Affiliations:** ^1^School of Management, Beijing University of Chinese Medicine, Beijing, China; ^2^School of Traditional Chinese Medicine, Beijing University of Chinese Medicine, Beijing, China; ^3^School of Computer Science and Statistics, Trinity College, Dublin, Ireland; ^4^Internal Medicine Department, Kaifeng Hospital of Traditional Chinese Medicine, Kaifeng, Henan, China

**Keywords:** COVID-19, self-leadership, risk perception, quality of life, self-punishment

## Abstract

**Background:**

Self-leadership has proven to adjust individual psychological states and promote active behaviors to mitigate stress perception and negative lifestyle. This study aims to investigate the relationship between self-leadership, epidemic risk perception, and quality of life among the general public in post-pandemic mainland of China.

**Methods:**

Two online self-reported questionnaire surveys were carried out with 3,098 and 469 people in the Chinese mainland in February 2021 and December 2022, respectively. The univariate analysis, structural equation modeling, and fuzzy-set qualitative comparative analysis were used to analyze the data which was collected by Revised Self-Leadership Questionnaire, Perceived Risk of COVID-19 Pandemic Scale and World Health Organization Quality of Life Brief Scale.

**Results:**

The Self-leadership was directly, moderately, and positively correlated with quality of life (Standardized path coefficients: 0.383 and 0.491, respectively; *p* < 0.05), and epidemic risk perception was negatively correlated with quality of life (Standardized path: 0.068 and 0.120, respectively; *p* < 0.05). The structural equation model for self-leadership, epidemic risk perception, and quality of life had a good fit (CFI = 0.957, 0.939 > 0.9; RSMEA = 0.058, 0.064 < 0.08, respectively) and was consistent across genders, educational levels, and types of occupations (Delata-CFI < 0.01). The core condition for achieving a high quality of life lies in maintaining a low level of self-punishment and a high level of self-cueing or a high level of self-punishment and a low level of self-cueing.

**Conclusion:**

In the post-epidemic era, the public can adjust their attitude toward stress by enhancing their self-leadership skills. Among various self-leadership skills, self-punishment or self-cueing may have the most significant impact on the quality of life.

## Introduction

The coronavirus disease 2019 (COVID-19) is an infectious disease that mainly damages the respiratory system and has significant physical, psychological, and social implications for people (1). Since the first case of COVID-19 was reported in Wuhan, China in December 2019 (2), more than 762 million confirmed cases and 6.8 million deaths worldwide have been reported by the World Health Organization (WHO) as of April 8, 2023 (3). China adopted proactive control policies in June 2020 to fight COVID-19 and has entered the post-pandemic era (4, 5) with the majority of the population vaccinated (6) and a lower fatality rate than before (7). Studies in various countries (8–10) revealed that people’s psychological stress under COVID-19 directly affected their quality of life and work performance. Therefore, exploring the various factors that affect people’s psychological state and behavior in the post-pandemic era can help to directly or indirectly improve their quality of life.

Self-leadership is a process of self-influence based on positive psychology to achieve target performance through self-guidance and motivation, as well as a derivative of self-management (11). Through self-leadership, individuals/groups adopt positive behaviors to achieve goals. In entrepreneurial teams, self-leadership is widely used to improve team performance and competitiveness (12). In the field of education, self-leadership is used to improve teaching quality and promote the improvement of student achievement.

Self-leadership (13) encompasses three primary strategies: behavior-focused strategies, natural reward strategies, and constructive thought pattern strategies, along with nine dimensions: self-observation, self-goal setting, self-reward, self-punishment, self-cueing, natural reward, visualizing successful performance, self-talk, and evaluation of beliefs and assumptions.

### Behavior-focused strategy

Behavior-focused involves the enhancing and improving self-awareness through self-evaluation and self-regulation to motivate positive and effective behaviors while suppress negative or ineffective ones. This strategy includes:

(1) Self-observation: Self-observation is the foundation for self-evaluation and self-goal setting. It requires attention to the timing, reasons, context, and manner of one’s behavior. By reviewing the circumstances and actions taken during past events or difficulties, one gains more understanding of their behavior. This self-awareness is the first step in identifying effective behaviors and problem-solving.
(2) Self-goal setting: Self-goal setting is an effective guide for self-behavior management. Setting reasonable goals can quickly and effectively influence and manage an individual’s behavior. Challenging goals can significantly increase individual performance or action levels but may also bring substantial negative impacts.
(3) Self-reward: Self-reward is one of the effective ways to enhance self-leadership. It encourages individuals to actively achieve their goals. Such rewards can be material or spiritual, such as a sumptuous dinner or a short trip. Note that these rewards should be implemented after the completion of the set goals.
(4) Self-punishment: The impact model of self-punishment is similar to that of self-reward. Self-punishment generates self-corrective feedback that leads to introspection and correction of failures and unpleasant behaviors, thus influencing behavior toward positive expected outcomes. However, excessive use of self-punishment can generate negative emotions that affect the expected results, as intense guilt and frequent self-denial can severely impact an individual’s motivation and creativity.
(5) Self-cueing: Self-cueing is an effective method for individuals to engage in positive behaviors and avoid negative ones to achieve set goals. It includes both positive and negative cues. For example, high-calorie diets in weight loss are negative cues, while a weighing scale is a positive cue. Avoiding negative cues and actively establishing positive ones are effective ways to reach set goals.

### Natural reward strategy

Natural reward refers to the process of enhancing self-control and achieving goals by creating and discovering fun and pleasure in a given task or behavior. Natural reward allows individuals to shift attention away from the uninteresting aspects of a task and refocus on the rewarding aspects inherent in the task itself, forming a perception of the activity through focusing on the intrinsic motivation of the work.

### Constructive thought pattern strategy

Constructive thought patterns involve reshaping an individual’s primary mental processes to achieve a more positive, optimistic, and upward-looking mindset, ultimately influencing the process of individual behavior. This strategy mainly includes:

(1) Visualizing successful performance: Success visualization is the process of imagining oneself succeeding before the actual success. The study has shown that people who rehearse and imagine themselves performing successfully in a task have a higher chance of success than those who start by imagining failure or negative outcomes (14).
(2) Self-talk: Self-talk refers to the process of engaging in positive internal dialogue. Individuals can effectively reduce and eliminate negative, irrational, and pessimistic dialogue by focusing on positive self-affirmations and self-motivation, encouraging more positive dialogue content, thus reducing anxiety and helping to overcome difficulties.
(3) Evaluation of beliefs and assumptions: Belief and assumption evaluation involves analyzing the accuracy of one’s current beliefs and the extent to which they influence one’s thinking and behavior, and whether they have a positive effect.

In the post-epidemic period, the widespread epidemic has brought challenges to the orderly management of government departments and work units, and Forbes et al. (15) have shown that people with certain self-management capabilities and a positive attitude can adapt to the adverse effects brought about by the epidemic more rapidly. Unlike the “top-down” training management and stimulation process of leadership, self-leadership highlights that via specific behavioral cognitive strategies, individuals can stimulate self-guidance and self-motivation to adapt to the environment and enhance internal driving forces (16).

Epidemic risk perception is an individual’s subjective perception of whether they can be infected with infectious diseases. The stronger the risk perception, the more they think that they are more likely to be infected with diseases. Studies have shown that the epidemic risk perception of the COVID-19 could positively predict Generalized Anxiety Disorder (17, 18), and even stress caused by the same stimulus in different situations will have different effects to individuals. Understanding the epidemic risk perception of people in a timely manner can provide a basis for the formulation of prevention and control measures, and can also provide evidence for how to regulate the risk perception of people. At present, there are barely any relevant studies on the impact of self-leadership on quality of life (19) and only a few studies on the impact of epidemic risk perception on quality of life (20). Hence, this study aims to explore the influence of self-leadership and epidemic risk perception on quality of life based on two surveys of Chinese mainland citizens in early 2021 and late 2022 (when the epidemic control and prevention measures were fully implemented) to examine the effect of individual active behavior on improving quality of life in public health emergencies.

## Methods

### Study design

In the cross-sectional surveys, convenient sampling and snowball sampling methods were used to recruit people in mainland of China, and electronic questionnaires were designed using the “So Jump” online platform^1^ (21). WeChat and QQ, the most widely used instant messaging tools in mainland of China, were used to distribute questionnaires from February to March 2021 and from December 2022 to January 2023. All the participants signed informed digital consent and completely submitted the electronic questionnaire only after filling out the informed consent and all items.

**Figure 1:**
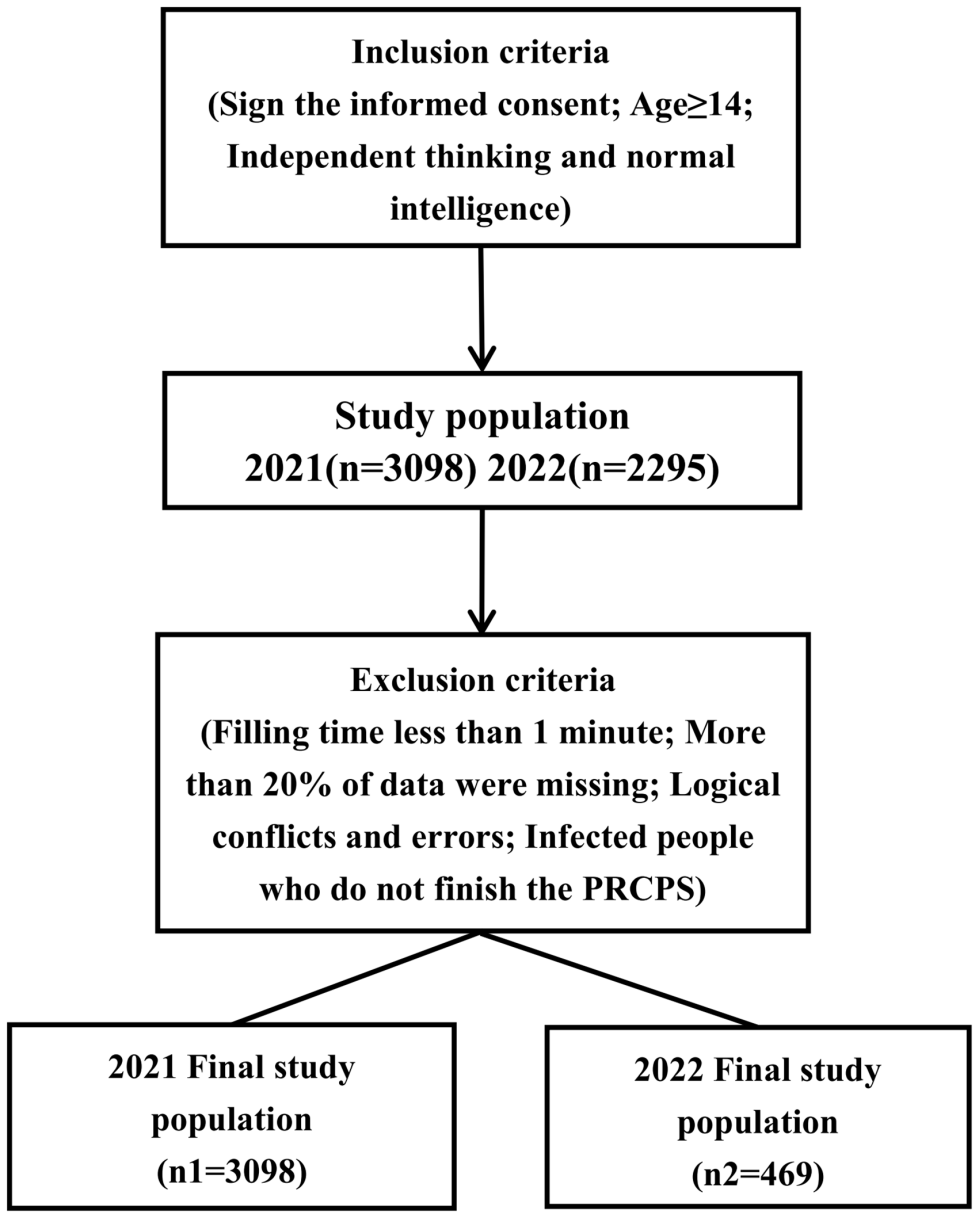
The flow diagram of participants in study.

The inclusion criteria are as follows: (a) People who will voluntarily participate and sign the informed consent prior to the survey; (b) People who are at least 14 years old (people aged 14 are usually regarded as having civil responsibility in China) and have independent thinking and normal intelligence to answer questions. The exclusion criteria are as follows: (a) Responses with short filling time (less than 1 min) and missing large amount of data (more than 20% of data were missing); (b) According to experts in the field of health, there are logical conflicts and errors in the contents; (c) People who do not finish the PRCPS [In the 2022 survey, only residents who are not infected with COVID-19 (tested negative in the nucleic acid or antigen test) fill out the questionnaire]. Finally, the two surveys preliminarily collect the responses of 3,098 and 2,295 people, respectively. After excluding invalid questionnaires, there remained 3,098 and 469 responses, respectively and the researchers could not identify the participants through the data (Figure 1).

### Measurements and instruments

#### Revised Self-Leadership Questionnaire (RSLQ)

The RSLQ is a 9-dimensional subjective questionnaire to measure self-leadership, translated into Chinese by Wen Wang (22). It includes 35 items for three strategies: behavior-focused strategies (self-observation, self-goal setting, self-reward, self-punishment and self-cueing), natural reward strategies (natural reward), and constructive thought pattern strategies (visualizing successful performance, self-talk, evaluation beliefs and assumptions). The items are scored on a 5-point Likert scale, with the total score ranging from 35 to 175 and a higher score indicating stronger self-leadership. The 9-dimension Cronbach’s coefficients for the present surveys were 0.718–0.884 and 0.589–0.881, and the total score coefficients were 0.976 and 0.960.

### Perceived risk of COVID-19 pandemic scale (PRCPS)

The PRCPS developed by Xi and colleagues (23) consists of 9 items scored on different Likert scales (Item 1 is measured on a 1–5 grade; Items 3, 5, and 9 are graded from 1 to 4; Items 2, 4, 6, 7, and 8 are graded from 1 to 6). The total score ranges from 9 to 47 with a higher score indicating a higher degree of epidemic risk perception. The instrument has good internal consistency, construct validity, and criterion validity. The Cronbach’s coefficient for the present surveys were 0.793 and 0.850.

#### World Health Organization Quality of Life Brief Scale (WHOQOL-BREF)

The Chinese version of the WHOQOL-BREF translated by Fang and colleagues has shown good internal consistency, discriminant validity, and construct validity (24). The instrument has 26 items which, graded on a 5-point Likert scale of 1–5, cover two items on general health evaluation and four dimensions: physical health (7 items), psychological well-being (6 items), social relationships (3 items), and environment (8 items). The total converted points range from 0 to 100, with more points reflecting a better quality of life. The 4-dimension Cronbach’s coefficients for the present surveys were 0.671–0.862 and 0.633–0.835, and the total score coefficients were 0.921 and 0.909.

### Statistical analyses

The Kolmogorov–Smirnov test was used to evaluate the normality of continuous variables and bivariate Pearson correlation analysis (based on approximately continuous normal data) was used to examine the correlations among self-leadership, epidemic risk perception, and quality of life. Subsequently, a structural equation model was developed to analyze the relationship among the variables and the effect of gender, education level, and occupation on this association. The total score of WHOQOL-BREF was taken as the dependent variable and the average scores of nine dimensions of RSLQ as the independent variable in the fuzzy-set qualitative comparative analysis (fsQCA) that adjusted the scores of the nine dimensions of self-leadership and the total scores of quality of life by “0–1” membership degree scores (25). To calibrate the fuzzy sets, the average scores of nine dimensions of self-leadership were used to set full non-membership, cross-over point, and full membership. The average of the total quality of life scores’ full non-membership, crossover, and full membership was Mean-2 Standard deviation, Mean, and Mean + 2 Standard deviation (Full non-membership, cross-over point and full membership represent 0.05, 0.5 and 0.95, respectively; and the closer the adjusted total quality of life score is to 1, the better the quality of life tends to be, while the non-set of the quality of life is the opposite in the study). The minimum number of effective cases was set at more than 1.00% of the number of cases. All the data were input and analyzed using IBM SPSS version 23.0, Amos version 24.0, and fsQCA version 3.0 for Windows.

## Results

### Sample characteristics

The sample characteristics of participants and the total scores of WHOQOL-BREF, PRCPS, and RSLQ are summarized in Table 1. About 40% (40.09, 42.00%) of the two surveys were male, and the averages of ages were (29.83 ± 10.42) and (25.94 ± 9.63), respectively. The basic characteristics of the participants and the levels of self-leadership are similar in the two surveys [total scores: (119.17 ± 26.29), (121.25 ± 21.90)]. The epidemic risk perception of participants was significantly higher in 2022 (31.01 ± 6.72) than in 2021 (20.77 ± 5.70), whereas the quality of life showed the opposite trend [total scores: (62.62 ± 14.50), (60.30 ± 14.16)], with the independent sample *T*-test showing significant differences (two-tailed, *p* < 0.05).

**Table 1:** Characteristics of participants.

	2021 study sample	2022 study sample
Variables	*N* = 3,098	*N* = 469
Male, n (%)	1,242 (40.09)	197 (42.00)
Age, mean (SD)	29.83 ± 10.42	25.94 ± 9.63
Educational level, n (%)		
Associate degree or below	1,138 (36.73)	131 (27.93)
Bachelor’ s degree	1,281 (41.35)	220 (46.91)
Master’ s degree or above	679 (21.92)	118 (25.16)
Occupation, *n* (%)		
Medical practitioner	483 (15.59)	34 (7.25)
Non-medical practitioner	2,615 (84.41)	435 (92.75)
Total score of QoL scale, mean (SD)	62.62 ± 14.50	60.30 ± 14.16
Total score of PRCPS, mean (SD)	20.77 ± 5.70	31.01 ± 6.72
Total score of RSLQ, mean (SD)	119.17 ± 26.29	121.25 ± 21.90

### Correlation between self-leadership, epidemic risk perception, and quality of life

The Pearson correlation coefficient was used to analyze the relationship between participants’ self-leadership, epidemic risk perception, and quality of life. The results of the correlation analysis are shown in Tables 2, 3. All the variables met the normality condition. Statistically significant positive correlations were found between self-leadership and epidemic risk perception in both surveys (*r* = 0.046, *p* < 0.05; *r* = 0.116, *p* < 0.05), and the correlation became stronger with the spread of the disease. “Self-goal setting,” “self-cueing,” and “natural reward” had no significant correlation with epidemic risk perception (Table 2). All the dimensions and the total of self-leadership could significantly improve the quality of life (*r* > 0, *p* < 0.01), with “self-punishment” having the least effect. As Table 3 shows, “self-cueing” and “evaluation beliefs and assumptions” had a less significant influence on epidemic risk perception. In addition, apart from “self-punishment” which had an insignificant positive influence on the “social relationships” and “physical health” aspects of quality of life, the other eight dimensions of self-leadership had significant effects on the four dimensions and total scores of quality of life (*r* > 0, *p* < 0.05).

**Table 2:** Pearson coefficient correlations between the self-leadership, risk perception and QoL (*n* = 3,098).

	1	2	3	4	5	6	7	8	9	10	11	12	13	14	15	16
Total scores of PRCPS	1	−0.192^**^	−0.183^**^	−0.164^**^	−0.107^**^	−0.182^**^	0.017	0.041^*^	0.046^*^	0.030	0.024	0.050^**^	0.037^*^	0.047^**^	0.088^**^	0.046^*^
PHYS (QoL)		1	0.734^**^	0.684^**^	0.649^**^	0.868^**^	0.308^**^	0.257^**^	0.225^**^	0.194^**^	0.295^**^	0.224^**^	0.296^**^	0.219^**^	0.105^**^	0.269^**^
PSYCH (QoL)			1	0.725^**^	0.666^**^	0.892^**^	0.356^**^	0.296^**^	0.279^**^	0.240^**^	0.337^**^	0.273^**^	0.334^**^	0.271^**^	0.127^**^	0.317^**^
ENVIR (QoL)				1	0.674^**^	0.879^**^	0.325^**^	0.289^**^	0.283^**^	0.236^**^	0.321^**^	0.277^**^	0.312^**^	0.273^**^	0.158^**^	0.311^**^
SOCIL (QoL)					1	0.863^**^	0.275^**^	0.226^**^	0.212^**^	0.184^**^	0.261^**^	0.238^**^	0.267^**^	0.203^**^	0.111^**^	0.249^**^
Total scores of QoL						1	0.360^**^	0.304^**^	0.285^**^	0.244^**^	0.346^**^	0.289^**^	0.344^**^	0.275^**^	0.143^**^	0.327^**^
Self-goal setting (RSLQ)							1	0.881^**^	0.766^**^	0.699^**^	0.877^**^	0.792^**^	0.877^**^	0.842^**^	0.741^**^	0.940^**^
Self-observation (RSLQ)								1	0.756^**^	0.693^**^	0.888^**^	0.780^**^	0.871^**^	0.834^**^	0.786^**^	0.938^**^
Self-reward (RSLQ)									1	0.605^**^	0.771^**^	0.727^**^	0.799^**^	0.740^**^	0.682^**^	0.851^**^
Self-cueing (RSLQ)										1	0.670^**^	0.641^**^	0.657^**^	0.674^**^	0.582^**^	0.756^**^
Natural reward (RSLQ)											1	0.782^**^	0.879^**^	0.839^**^	0.769^**^	0.941^**^
Self-talk (RSLQ)												1	0.796^**^	0.781^**^	0.695^**^	0.869^**^
Evaluation beliefs and assumptions (RSLQ)													1	0.833^**^	0.763^**^	0.937^**^
Visualizing successful performance (RSLQ)														1	0.744^**^	0.917^**^
Self-punishment (RSLQ)															1	0.845^**^
Total scores of RSLQ																1

PHYS, Physical health; PSYCH, Psychological well-being; ENVIR, Environment; SOCIL, Social relationships; ^*^p < 0.05 (double-tailed); ^**^p < 0.01 (double-tailed); all based on Pearson’s coefficient correlations.

**Table 3:** Pearson coefficient correlations between the self-leadership, risk perception and QoL (*n* = 469).

	1	2	3	4	5	6	7	8	9	10	11	12	13	14	15	16
Total scores of PRCPS	1	−0.133^**^	−0.076	−0.084	−0.028	−0.090	0.123^**^	0.113^*^	0.109^*^	0.049	0.093^*^	0.091^*^	0.078	0.096^*^	0.106^*^	0.116^*^
PHYS (QoL)		1	0.676^**^	0.627^**^	0.636^**^	0.847^**^	0.341^**^	0.285^**^	0.143^**^	0.130^**^	0.321^**^	0.265^**^	0.326^**^	0.227^**^	0.016	0.282^**^
PSYCH (QoL)			1	0.677^**^	0.622^**^	0.862^**^	0.417^**^	0.349^**^	0.226^**^	0.214^**^	0.392^**^	0.373^**^	0.429^**^	0.333^**^	0.105^*^	0.384^**^
ENVIR (QoL)				1	0.675^**^	0.861^**^	0.410^**^	0.376^**^	0.320^**^	0.241^**^	0.407^**^	0.365^**^	0.402^**^	0.354^**^	0.188^**^	0.412^**^
SOCIL (QoL)					1	0.867^**^	0.328^**^	0.295^**^	0.192^**^	0.185^**^	0.348^**^	0.317^**^	0.322^**^	0.214^**^	0.072	0.306^**^
Total scores of QoL						1	0.433^**^	0.378^**^	0.255^**^	0.224^**^	0.426^**^	0.384^**^	0.428^**^	0.325^**^	0.110^*^	0.401^**^
Self-goal setting (RSLQ)							1	0.811^**^	0.644^**^	0.620^**^	0.805^**^	0.710^**^	0.833^**^	0.748^**^	0.590^**^	0.906^**^
Self-observation (RSLQ)								1	0.604^**^	0.596^**^	0.809^**^	0.693^**^	0.829^**^	0.739^**^	0.659^**^	0.897^**^
Self-reward (RSLQ)									1	0.453^**^	0.600^**^	0.530^**^	0.686^**^	0.630^**^	0.564^**^	0.759^**^
Self-cueing (RSLQ)										1	0.554^**^	0.523^**^	0.586^**^	0.562^**^	0.443^**^	0.683^**^
Natural reward (RSLQ)											1	0.689^**^	0.846^**^	0.761^**^	0.665^**^	0.903^**^
Self-talk (RSLQ)												1	0.749^**^	0.657^**^	0.513^**^	0.801^**^
Evaluation beliefs and assumptions (RSLQ)													1	0.785^**^	0.654^**^	0.930^**^
Visualizing successful performance (RSLQ)														1	0.611^**^	0.874^**^
Self-punishment (RSLQ)															1	0.755^**^
Total scores of RSLQ																1

PHYS, Physical health; PSYCH, Psychological well-being; ENVIR, Environment; SOCIL, Social relationships; ^*^p < 0.05 (double-tailed); ^**^p < 0.01 (double-tailed); all based on Pearson’s coefficient correlations.

### Structural equation analysis of self-leadership, epidemic risk perception, and quality of life

Based on the results of the univariate correlation analysis, the following hypotheses were proposed — H0: Self-leadership positively promotes the quality of life; H1: Self-leadership positively affects epidemic risk perception; H2: Epidemic risk perception has a negative impact on quality of life.

**Figure 2:**
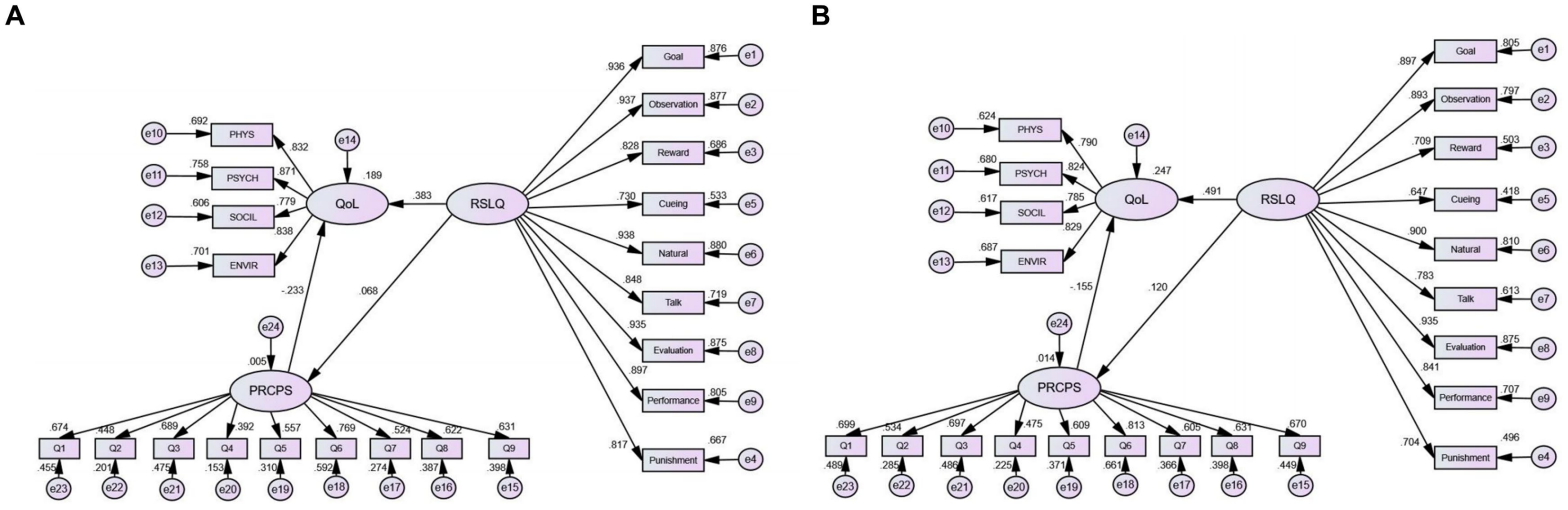
The structural equation in the self-leadership, risk perception and quality of life. **(A)** Relationship in 2021. **(B)** Relationship in 2022. PHYS, Physical health; PSYCH, Psychological well-being; ENVIR, Environment; SOCIL, Social relationships; Goal, Self-goal setting; Observation, Self-observation; Reward, Self-reward; Cueing, Self-cueing; Natural, Natural reward; Talk, Self-talk; Evaluation, Evaluation beliefs and assumptions; Performance, Visualizing successful performance; Punishment, Self-punishment.

Figure 2 shows the structural equation model with a comparison of the relationships between self-leadership, epidemic risk perception, and quality of life in 2021 and 2022. The fit indexes in 2021 were as follows: CMIN/DF = 11.374, TLI = 0.952 > 0.9, CFI = 0.957 > 0.9, and RSMEA = 0.058 < 0.08; the fit indexes in 2022 were as follows: CMIN/DF = 2.914, TLI = 0.932 > 0.9, CFI = 0.939 > 0.9, RSMEA = 0.064 < 0.08 and the path coefficients of the model were significant (*p* < 0.05). These indicated that the model had good fit and structural validity, confirming H0, H1, and H2 (*p* < 0.001) and showing that self-leadership could directly promote the quality of life and mitigate epidemic risk perception both during the pandemic and in the post-pandemic era. To further examine whether the above relationship held true for different populations and whether the model was stable across groups, we analyzed the relatively stable sociodemographic indicators, namely gender (male and female), education level (associate degree or below, bachelor’s degree, master’s degree or above), and occupation (medical practitioner or not) with controlling the other variables. The results of multi-group analysis based on gender, education level, and occupation in 2021 and 2022 are shown in Tables 4–6, respectively, indicating that the above relationships among different populations are still valid and stable, but with some differences. The standardized path coefficients of the model are reported in Tables 7–12.

**Table 4:** Invariance test in genders.

Year (case)	Model	CMIN/DF	*p*	TLI	CFI	RMSEA
	Unconstrained	6.386	***	0.950	0.955	0.042
	Comparative model		*p*	Delata-TLI	Delata-CFI	Delata-RMSEA
2021 (3098)	Measurement weights	6.216	***	0	−0.001	−0.001
	Structural weights	6.192	***	0	−0.001	−0.001
	Structural covariances	6.269	***	−0.001	−0.003	−0.001
	Structural residuals	6.278	***	−0.001	−0.003	−0.001
	Measurement residuals	6.206	***	−0.003	−0.006	−0.001

	Unconstrained	2.128	***	0.920	0.929	0.049
	Comparative model		*p*	Delata-TLI	Delata-CFI	Delata-RMSEA
2022 (469)	Measurement weights	2.084	0.332	0.003	−0.001	−0.001
	Structural weights	2.079	0.290	0.004	−0.001	−0.001
	Structural covariances	2.079	0.244	0.003	−0.001	−0.001
	Structural residuals	2.070	0.320	0.004	−0.001	−0.001
	Measurement residuals	2.153	***	−0.002	−0.010	0.001

***p < 0.001 (double-tailed); When the *p* value is < 0.05 and the absolute values of Delata-TLI, Delata-CFI, and Delata-RMSEA exceed 0.05, significant multi-group differences can be considered, as demonstrated in Tables 5, 6.

**Table 5:** Invariance test in education levels.

Year (case)	Model	CMIN/DF	*p*	TLI	CFI	RMSEA
	Unconstrained	4.717	***	0.950	0.955	0.042
	Comparative model		*p*	Delata-TLI	Delata-CFI	Delata-RMSEA
2021 (3098)	Measurement weights	4.527	0.038	0.003	−0.001	−0.001
	Structural weights	4.497	0.035	0.003	−0.001	−0.001
	Structural covariances	4.580	***	0.002	−0.002	−0.001
	Structural residuals	4.567	***	0.002	−0.002	−0.001
	Measurement residuals	4.450	***	0.004	−0.004	−0.002

	Unconstrained	1.896	***	0.920	0.929	0.049
	Comparative model		*p*	Delata-TLI	Delata-CFI	Delata-RMSEA
2022 (469)	Measurement weights	1.865	0.729	0.003	0	−0.001
	Structural weights	1.860	0.763	0.004	0	−0.001
	Structural covariances	1.859	0.723	0.004	0	−0.001
	Structural residuals	1.859	0.636	0.004	0	−0.001
	Measurement residuals	1.843	0.294	0.005	−0.001	−0.001

**Table 6:** Invariance test in occupations.

Year (case)	Model	CMIN/DF	*p*	TLI	CFI	RMSEA
	Unconstrained	6.382	***	0.950	0.956	0.042
	Comparative model		*p*	Delata-TLI	Delata-CFI	Delata-RMSEA
2021 (3098)	Measurement weights	6.157	0.187	0.002	−0.001	−0.001
	Structural weights	6.115	0.318	0.003	−0.001	−0.001
	Structural covariances	6.215	***	0.002	−0.001	−0.001
	Structural residuals	6.191	***	0.002	−0.001	−0.001
	Measurement residuals	6.091	***	0.003	−0.003	−0.001

	Unconstrained	2.392	***	0.920	0.929	0.049
	Comparative model		*p*	Delata-TLI	Delata-CFI	Delata-RMSEA
2022 (469)	Measurement weights	2.381	0.003	0	−0.003	−0.001
	Structural weights	2.378	0.002	0.001	−0.004	−0.001
	Structural covariances	2.379	0.001	0.001	−0.004	−0.001
	Structural residuals	2.369	0.002	0.001	−0.004	−0.001
	Measurement residuals	2.330	***	0.004	−0.005	−0.002

**Table 7:** The standardized path coefficients of the model in 2021 (genders).

Year	Latent variable		Observation variable	Unstandardized estimate	Standardized estimate	S.E.	*t*-value	*p*
				Male (Female)				
2021	RSLQ	→	PRCPS	0.003 (0.012)	0.028 (0.090)	0.003 (0.004)	0.883 (3.505)	0.377 (***)
	RSLQ	→	QoL	1.386 (1.321)	0.380 (0.380)	0.103 (0.084)	13.525 (15.721)	*** (***)
	PRCPS	→	QoL	−8.519 (−5.401)	−0.259 (−0.216)	1.036 (0.643)	−8.223 (−8.398)	*** (***)
	QoL	→	ENVIR (QoL)	1.000 (1.000)	0.871 (0.809)			
	QoL	→	SOCIL (QoL)	1.000 (1.071)	0.777 (0.781)	0.030 (0.029)	33.436 (36.784)	*** (***)
	QoL	→	PSYCH (QoL)	1.014 (1.125)	0.876 (0.866)	0.025 (0.027)	40.019 (41.259)	*** (***)
	QoL	→	PHYS (QoL)	0.893 (0.964)	0.850 (0.818)	0.023 (0.025)	38.255 (38.313)	*** (***)
	RSLQ	→	Self-goal setting (RSLQ)	1.000 (1.000)	0.941 (0.930)			
	RSLQ	→	Self-observation (RSLQ)	0.791 (0.771)	0.944 (0.929)	0.011 (0.010)	69.231 (75.429)	*** (***)
	RSLQ	→	Self-reward (RSLQ)	0.612 (0.610)	0.847 (0.811)	0.013 (0.012)	48.036 (51.426)	*** (***)
	RSLQ	→	Self-cueing (RSLQ)	0.357 (0.376)	0.746 (0.714)	0.010 (0.009)	36.222 (40.152)	*** (***)
	RSLQ	→	Natural reward (RSLQ)	1.001 (0.959)	0.944 (0.932)	0.014 (0.013)	69.172 (76.243)	*** (***)
	RSLQ	→	Self-talk (RSLQ)	0.600 (0.597)	0.862 (0.833)	0.012 (0.011)	50.458 (54.813)	*** (***)
	RSLQ	→	Evaluation beliefs and assumptions (RSLQ)	0.801 (0.801)	0.941 (0.929)	0.012 (0.011)	68.445 (75.354)	*** (***)
	RSLQ	→	Visualizing successful performance (RSLQ)	0.970 (0.937)	0.921 (0.876)	0.015 (0.015)	62.640 (62.384)	*** (***)
	RSLQ	→	Self-punishment (RSLQ)	0.669 (0.643)	0.837 (0.796)	0.014 (0.013)	46.485 (49.404)	*** (***)
	PRCPS	→	Q9 (PRCPS)	1.000 (1.000)	0.598 (0.652)			
	PRCPS	→	Q8 (PRCPS)	0.936 (0.990)	0.588 (0.643)	0.054 (0.040)	17.499 (24.662)	*** (***)
	PRCPS	→	Q7 (PRCPS)	0.983 (0.855)	0.538 (0.521)	0.063 (0.044)	15.514 (19.597)	*** (***)
	PRCPS	→	Q6 (PRCPS)	1.430 (1.395)	0.758 (0.774)	0.077 (0.054)	18.473 (25.830)	*** (***)
	PRCPS	→	Q5 (PRCPS)	1.803 (1.617)	0.553 (0.560)	0.119 (0.008)	15.168 (20.290)	*** (***)
	PRCPS	→	Q4 (PRCPS)	0.970 (1.253)	0.305 (0.454)	0.104 (0.073)	9.329 (17.160)	*** (***)
	PRCPS	→	Q3 (PRCPS)	1.474 (1.365)	0.676 (0.696)	0.086 (0.057)	17.189 (24.064)	*** (***)
	PRCPS	→	Q2 (PRCPS)	1.047 (1.075)	0.411 (0.469)	0.085 (0.061)	12.373 (17.769)	*** (***)
	PRCPS	→	Q1 (PRCPS)	1.074 (0.899)	0.684 (0.667)	0.062 (0.039)	17.372 (23.191)	*** (***)

^***^p< 0.001 (double-tailed);as demonstrated in Tables 8–12.

**Table 8:** The standardized path coefficients of the model in 2022 (genders).

Year	Latent variable		Observation variable	Unstandardized estimate	Standardized estimate	S.E.	*t*-value	*p*
				Male (Female)				
2022	RSLQ	→	PRCPS	0.011 (0.023)	0.059 (0.137)	0.014 (0.011)	0.755 (2.061)	0.450 (0.039)
	RSLQ	→	QoL	1.786 (2.099)	0.467 (0.502)	0.292 (0.265)	6.113 (7.910)	*** (***)
	PRCPS	→	QoL	−4.900 (−2.307)	−0.227 (−0.093)	1.641 (1.544)	−2.986 (−1.494)	0.003 (0.135)
	QoL	→	ENVIR (QoL)	1.000 (1.000)	0.819 (0.834)			
	QoL	→	SOCIL (QoL)	1.170 (1.161)	0.769 (0.799)	0.101 (0.077)	11.586 (14.986)	*** (***)
	QoL	→	PSYCH (QoL)	1.094 (1.052)	0.818 (0.833)	0.089 (0.068)	12.245 (15.486)	*** (***)
	QoL	→	PHYS (QoL)	0.916 (0.973)	0.789 (0.792)	0.080 (0.068)	11.509 (14.402)	*** (***)
	RSLQ	→	Self-goal setting (RSLQ)	1.000 (1.000)	0.876 (0.911)			
	RSLQ	→	Self-observation (RSLQ)	0.806 (0.751)	0.919 (0.869)	0.041 (0.034)	19.572 (22.051)	*** (***)
	RSLQ	→	Self-reward (RSLQ)	0.642 (0.562)	0.766 (0.641)	0.046 (0.045)	14.000 (12.539)	*** (***)
	RSLQ	→	Self-cueing (RSLQ)	0.366 (0.356)	0.646 (0.633)	0.035 (0.029)	10.573 (12.357)	*** (***)
	RSLQ	→	Natural reward (RSLQ)	0.961 (0.936)	0.893 (0.901)	0.052 (0.039)	18.358 (24.003)	*** (***)
	RSLQ	→	Self-talk (RSLQ)	0.617 (0.589)	0.817 (0.749)	0.040 (0.036)	15.299 (16.181)	*** (***)
	RSLQ	→	Evaluation beliefs and assumptions (RSLQ)	0.854 (0.844)	0.942 (0.928)	0.041 (0.032)	20.813 (26.134)	*** (***)
	RSLQ	→	Visualizing successful performance (RSLQ)	0.952 (0.972)	0.862 (0.830)	0.056 (0.049)	17.003 (19.737)	*** (***)
	RSLQ	→	Self-punishment (RSLQ)	0.654 (0.537)	0.802 (0.622)	0.044 (0.045)	14.775 (11.951)	*** (***)
	PRCPS	→	Q9 (PRCPS)	1.000 (1.000)	0.661 (0.655)			
	PRCPS	→	Q8 (PRCPS)	0.919 (1.052)	0.607 (0.628)	0.119 (0.114)	7.733 (9.258)	*** (***)
	PRCPS	→	Q7 (PRCPS)	0.867 (0.875)	0.594 (0.601)	0.121 (0.100)	7.190 (8.760)	*** (***)
	PRCPS	→	Q6 (PRCPS)	1.705 (1.836)	0.810 (0.825)	0.190 (0.167)	8.978 (10.985)	*** (***)
	PRCPS	→	Q5 (PRCPS)	1.592 (1.499)	0.647 (0.589)	0.212 (0.177)	7.508 (8.462)	*** (***)
	PRCPS	→	Q4 (PRCPS)	0.621 (1.321)	0.307 (0.610)	0.159 (0.152)	3.901 (8.678)	*** (***)
	PRCPS	→	Q3 (PRCPS)	1.510 (1.609)	0.688 (0.699)	0.189 (0.164)	7.993 (9.822)	*** (***)
	PRCPS	→	Q2 (PRCPS)	1.127 (1.386)	0.471 (0.568)	0.190 (0.168)	5.930 (8.263)	*** (***)
	PRCPS	→	Q1 (PRCPS)	1.247 (1.349)	0.661 (0.733)	0.162 (0.134)	7.685 (10.081)	*** (***)

**Table 9:** The standardized path coefficients of the model in 2021 (education levels).

Year	Latent variable		Observation variable	Unstandardized estimate	Standardized estimate	S.E.	*t*-value	*p*
				Associate degree or below (Bachelor’ s degree/Master’s degree or above)				
2021	RSLQ	→	PRCPS	0.004 (0.010/0.006)	0.030 (0.077/0.055)	0.004 (0.004/0.005)	0.926 (2.500/1.302)	0.354 (0.012/0.193)
	RSLQ	→	QoL	1.523 (1.314/1.257)	0.400 (0.366/0.379)	0.116 (0.102/0.129)	13.122 (12.879/9.765)	*** (***/***)
	PRCPS	→	QoL	−5.773 (−7.390/−6.016)	−0.203 (−0.272/−0.204)	0.923 (0.854/1.238)	−6.254 (−8.654/−4.861)	***(***/***)
	QoL	→	ENVIR (QoL)	1.000 (1.000/1.000)	0.822 (0.843/0.851)			
	QoL	→	SOCIL (QoL)	1.041 (1.027/1.051)	0.757 (0.782/0.805)	0.037 (0.031/0.042)	28.284 (32.680/25.115)	*** (***/***)
	QoL	→	PSYCH (QoL)	1.064 (1.098/1.034)	0.871 (0.878/0.856)	0.032 (0.026/0.038)	33.144 (38.118/27.198)	*** (***/***)
	QoL	→	PHYS (QoL)	0.939 (0.953/0.873)	0.820 (0.836/0.843)	0.030 (0.027/0.033)	30.985 (35.392/26.360)	*** (***/***)
	RSLQ	→	Self-goal setting (RSLQ)	1.000 (1.000/1.000)	0.923 (0.932/0.950)			
	RSLQ	→	Self-observation (RSLQ)	0.755 (0.783/0.803)	0.919 (0.931/0.958)	0.014 (0.012/0.014)	55.188 (63.474/57.875)	*** (***/***)
	RSLQ	→	Self-reward (RSLQ)	0.621 (0.603/0.618)	0.799 (0.815/0.867)	0.016 (0.014/0.016)	38.405 (43.440/38.969)	*** (***/***)
	RSLQ	→	Self-cueing (RSLQ)	0.361 (0.378/0.368)	0.679 (0.722/0.792)	0.013 (0.011/0.012)	28.648 (34.082/30.783)	*** (***/***)
	RSLQ	→	Natural reward (RSLQ)	0.953 (0.993/0.996)	0.919 (0.938/0.955)	0.017 (0.015/0.018)	55.113 (65.443/56.899)	*** (***/***)
	RSLQ	→	Self-talk (RSLQ)	0.611 (0.597/0.588)	0.827 (0.841/0.870)	0.015 (0.013/0.015)	41.592 (46.768/39.429)	*** (***/***)
	RSLQ	→	Evaluation beliefs and assumptions (RSLQ)	0.796 (0.795/0.810)	0.923 (0.927/0.953)	0.014 (0.013/0.014)	55.935 (62.744/56.270)	*** (***/***)
	RSLQ	→	Visualizing successful performance (RSLQ)	0.951 (0.964/0.941)	0.884 (0.888/0.919)	0.019 (0.018/0.020)	49.124 (54.371/47.755)	*** (***/***)
	RSLQ	→	Self-punishment (RSLQ)	0.629 (0.664/0.678)	0.764 (0.816/0.863)	0.018 (0.015/0.018)	35.107 (43.505/38.377)	*** (***/***)
	PRCPS	→	Q9 (PRCPS)	1.000 (1.000/1.000)	0.624 (0.632/0.628)			
	PRCPS	→	Q8 (PRCPS)	1.002 (0.948/0.967)	0.628 (0.611/0.626)	0.055 (0.049/0.069)	18.270 (19.199/14.021)	*** (***/***)
	PRCPS	→	Q7 (PRCPS)	0.973 (0.857/0.842)	0.554 (0.504/0.501)	0.062 (0.055/0.076)	15.627 (15.600/11.144)	*** (***/***)
	PRCPS	→	Q6 (PRCPS)	1.360 (1.415/1.452)	0.759 (0.774/0.767)	0.073 (0.068/0.097)	18.509 (20.722/14.970)	*** (***/***)
	PRCPS	→	Q5 (PRCPS)	1.570 (1.690/1.780)	0.489 (0.575/0.617)	0.115 (0.101/0.138)	13.656 (16.768/12.856)	*** (***/***)
	PRCPS	→	Q4 (PRCPS)	1.268 (1.189/0.922)	0.428 (0.412/0.312)	0.104 (0.093/0.127)	12.228 (12.808/7.276)	*** (***/***)
	PRCPS	→	Q3 (PRCPS)	1.458 (1.461/1.295)	0.687 (0.715/0.661)	0.084 (0.074/0.097)	17.453 (19.652/13.313)	*** (***/***)
	PRCPS	→	Q2 (PRCPS)	1.018 (1.169/1.012)	0.437 (0.479/0.415)	0.081 (0.078/0.107)	12.630 (14.914/9.422)	*** (***/***)
	PRCPS	→	Q1 (PRCPS)	0.963 (0.944/1.014)	0.667 (0.675/0.681)	0.056 (0.050/0.074)	17.167 (18.799/13.662)	*** (***/***)

**Table 10:** The standardized path coefficients of the model in 2022 (education levels).

Year	Latent variable		Observation variable	Unstandardized estimate	Standardized estimate	S.E.	*t*-value	*p*
				Associate degree or below (Bachelor’ s degree/Master’ s degree or above)				
2022	RSLQ	→	PRCPS	0.020 (0.012/−0.001)	0.125 (0.060/−0.006)	0.011 (0.015/0.016)	1.774 (0.819/−0.055)	0.076 (0.413/0.956)
	RSLQ	→	QoL	1.846 (2.096/2.684)	0.469 (0.506/0.512)	0.269 (0.288/0.505)	6.874 (7.289/5.310)	*** (***/***)
	PRCPS	→	QoL	−2.489 (−4.305/–1.150)	−0.100 (−0.205/−0.035)	1.683 (1.444/3.008)	−1.479 (−2.980/−0.382)	0.139 (0.003/0.702)
	QoL	→	ENVIR (QoL)	1.000 (1.000/1.000)	0.827 (0.838/0.850)			
	QoL	→	SOCIL (QoL)	1.131 (1.183/1.151)	0.752 (0.812/0.821)	0.089 (0.085/0.104)	12.775 (13.840/11.031)	*** (***/***)
	QoL	→	PSYCH (QoL)	1.085 (1.053/1.111)	0.842 (0.810/0.882)	0.075 (0.079/0.098)	14.531 (13.365/11.350)	*** (***/***)
	QoL	→	PHYS (QoL)	0.919 (0.978/0.985)	0.768 (0.809/0.814)	0.073 (0.072/0.098)	12.542 (13.599/10.101)	*** (***/***)
	RSLQ	→	Self-goal setting (RSLQ)	1.000 (1.000/1.000)	0.897 (0.891/0.848)			
	RSLQ	→	Self-observation (RSLQ)	0.796 (0.740/0.677)	0.915 (0.858/0.821)	0.034 (0.040/0.062)	23.177 (18.322/11.001)	*** (***/***)
	RSLQ	→	Self-reward (RSLQ)	0.624 (0.568/0.589)	0.734 (0.662/0.628)	0.042 (0.049/0.078)	14.729 (11.585/7.535)	*** (***/***)
	RSLQ	→	Self-cueing (RSLQ)	0.377 (0.353/0.375)	0.681 (0.596/0.606)	0.029 (0.035/0.052)	13.067 (10.058/7.225)	*** (***/***)
	RSLQ	→	Natural reward (RSLQ)	1.003 (0.874/0.951)	0.912 (0.878/0.866)	0.043 (0.046/0.077)	23.091 (19.032/12.344)	*** (***/***)
	RSLQ	→	Self-talk (RSLQ)	0.626 (0.582/0.578)	0.806 (0.743/0.697)	0.036 (0.042/0.067)	17.450 (13.884/8.665)	*** (***/***)
	RSLQ	→	Evaluation beliefs and assumptions (RSLQ)	0.878 (0.821/0.879)	0.938 (0.933/0.918)	0.035 (0.037/0.066)	24.849 (22.081/13.302)	*** (***/***)
	RSLQ	→	Visualizing successful performance (RSLQ)	0.988 (0.909/1.005)	0.852 (0.819/0.772)	0.050 (0.055/0.100)	19.579 (16.511/10.067)	*** (***/***)
	RSLQ	→	Self-punishment (RSLQ)	0.635 (0.524/0.499)	0.727 (0.659/0.553)	0.044 (0.046/0.078)	14.419 (11.482/6.390)	*** (***/***)
	PRCPS	→	Q9 (PRCPS)	1.000 (1.000/1.000)	0.644 (0.691/0.601)			
	PRCPS	→	Q8 (PRCPS)	0.886 (1.056/0.884)	0.528 (0.722/0.507)	0.119 (0.106/0.185)	7.443 (9.95/4.771)	*** (***/***)
	PRCPS	→	Q7 (PRCPS)	0.920 (0.769/0.696)	0.587 (0.592/0.394)	0.116 (0.097/0.184)	7.948 (7.903/3.784)	*** (***/***)
	PRCPS	→	Q6 (PRCPS)	1.799 (1.612/2.324)	0.795 (0.813/0.835)	0.188 (0.156/0.363)	9.581 (10.324/6.400)	*** (***/***)
	PRCPS	→	Q5 (PRCPS)	1.669 (1.334/2.115)	0.617 (0.599/0.601)	0.208 (0.169/0.409)	8.019 (7.907/5.168)	*** (***/***)
	PRCPS	→	Q4 (PRCPS)	0.972 (0.964/1.810)	0.441 (0.500/0.640)	0.159 (0.144/0.322)	6.101 (6.683/5.623)	*** (***/***)
	PRCPS	→	Q3 (PRCPS)	1.628 (1.475/2.061)	0.665 (0.734/0.662)	0.192 (0.152/0.364)	8.482 (9.707/5.660)	*** (***/***)
	PRCPS	→	Q2 (PRCPS)	1.204 (1.325/1.623)	0.459 (0.609/0.505)	0.190 (0.160/0.354)	6.354 (8.284/4.585)	*** (***/***)
	PRCPS	→	Q1 (PRCPS)	1.404 (1.117/1.857)	0.668 (0.695/0.769)	0.160 (0.125/0.297)	8.772 (8.951/6.246)	*** (***/***)

**Table 11:** The standardized path coefficients of the model in 2021 (occupations).

Year	Latent variable		Observation variable	Unstandardized estimate	Standardized estimate	S.E.	*t*-value	*p*
				Medical practitioner (Non-medical practitioner)				
2021	RSLQ	→	PRCPS	0.009 (0.008)	0.063 (0.065)	0.007 (0.003)	1.255 (3.031)	0.209 (0.002)
	RSLQ	→	QoL	1.341 (1.367)	0.340 (0.390)	0.183 (0.070)	7.197 (19.640)	*** (***)
	PRCPS	→	QoL	−6.567 (−6.523)	−0.251 (−0.227)	1.357 (0.617)	−4.841 (−10.574)	*** (***)
	QoL	→	ENVIR (QoL)	1.000 (1.000)	0.796 (0.845)			
	QoL	→	SOCIL (QoL)	1.006 (1.034)	0.762 (0.781)	0.059 (0.022)	17.990 (46.584)	*** (***)
	QoL	→	PSYCH (QoL)	1.173 (1.055)	0.881 (0.868)	0.055 (0.020)	21.175 (53.351)	*** (***)
	QoL	→	PHYS (QoL)	1.026 (0.915)	0.844 (0.831)	0.051 (0.018)	20.099 (50.106)	*** (***)
	RSLQ	→	Self-goal setting (RSLQ)	1.000 (1.000)	0.915 (0.938)			
	RSLQ	→	Self-observation (RSLQ)	0.754 (0.782)	0.914 (0.939)	0.022 (0.008)	34.026 (96.840)	*** (***)
	RSLQ	→	Self-reward (RSLQ)	0.628 (0.613)	0.790 (0.833)	0.026 (0.009)	24.004 (66.289)	*** (***)
	RSLQ	→	Self-cueing (RSLQ)	0.365 (0.370)	0.670 (0.738)	0.020 (0.007)	18.061 (51.173)	*** (***)
	RSLQ	→	Natural reward (RSLQ)	0.934 (0.983)	0.916 (0.941)	0.027 (0.010)	34.375 (97.366)	*** (***)
	RSLQ	→	Self-talk (RSLQ)	0.576 (0.603)	0.775 (0.856)	0.025 (0.008)	23.241 (71.304)	*** (***)
	RSLQ	→	Evaluation beliefs and assumptions (RSLQ)	0.778 (0.802)	0.903 (0.939)	0.024 (0.008)	32.812 (96.728)	*** (***)
	RSLQ	→	Visualizing successful performance (RSLQ)	0.915 (0.955)	0.812 (0.908)	0.036 (0.011)	25.479 (85.282)	*** (***)
	RSLQ	→	Self-punishment (RSLQ)	0.646 (0.659)	0.760 (0.824)	0.029 (0.010)	22.270 (64.456)	*** (***)
	PRCPS	→	Q9 (PRCPS)	1.000 (1.000)	0.591 (0.634)			
	PRCPS	→	Q8 (PRCPS)	0.975 (0.978)	0.594 (0.627)	0.089 (0.035)	11.013 (27.949)	*** (***)
	PRCPS	→	Q7 (PRCPS)	0.986 (0.885)	0.560 (0.516)	0.099 (0.039)	9.968 (22.624)	*** (***)
	PRCPS	→	Q6 (PRCPS)	1.482 (1.393)	0.782 (0.764)	0.127 (0.048)	11.664 (29.211)	*** (***)
	PRCPS	→	Q5 (PRCPS)	1.695 (1.706)	0.537 (0.563)	0.181 (0.073)	9.377 (23.473)	*** (***)
	PRCPS	→	Q4 (PRCPS)	1.401 (1.101)	0.475 (0.375)	0.163 (0.066)	8.586 (16.799)	*** (***)
	PRCPS	→	Q3 (PRCPS)	1.661 (1.363)	0.751 (0.674)	0.144 (0.051)	11.497 (26.790)	*** (***)
	PRCPS	→	Q2 (PRCPS)	1.133 (1.082)	0.493 (0.445)	0.126 (0.055)	8.980 (19.807)	*** (***)
	PRCPS	→	Q1 (PRCPS)	0.934 (0.961)	0.589 (0.685)	0.094 (0.036)	9.939 (27.033)	*** (***)

**Table 12:** The standardized path coefficients of the model in 2022 (occupations).

Year	Latent variable		Observation variable	Unstandardized estimate	Standardized estimate	S.E.	*t*-value	*p*
				Medical practitioner (Non-medical practitioner)				
2022	RSLQ	→	PRCPS	0.085 (0.016)	0.550 (0.089)	0.032 (0.009)	2.675 (1.695)	0.007 (0.090)
	RSLQ	→	QoL	1.480 (1.970)	0.446 (0.494)	0.647 (0.201)	2.287 (9.789)	0.022 (***)
	PRCPS	→	QoL	6.168 (−3.691)	0.287 (−0.164)	3.730 (1.119)	1.654 (−3.299)	0.098 (***)
	QoL	→	ENVIR (QoL)	1.000 (1.000)	0.816 (0.823)			
	QoL	→	SOCIL (QoL)	0.311 (1.196)	0.211 (0.804)	0.274 (0.064)	1.136 (18.782)	0.256 (***)
	QoL	→	PSYCH (QoL)	1.626 (1.057)	1.051 (0.823)	0.318 (0.057)	5.112 (18.688)	*** (***)
	QoL	→	PHYS (QoL)	0.604 (0.962)	0.521 (0.798)	0.178 (0.054)	3.399 (17.955)	*** (***)
	RSLQ	→	Self-goal setting (RSLQ)	1.000 (1.000)	0.925 (0.896)			
	RSLQ	→	Self-observation (RSLQ)	0.924 (0.758)	0.951 (0.886)	0.085 (0.027)	10.876 (28.096)	*** (***)
	RSLQ	→	Self-reward (RSLQ)	0.708 (0.597)	0.845 (0.698)	0.094 (0.033)	7.568 (17.863)	*** (***)
	RSLQ	→	Self-cueing (RSLQ)	0.437 (0.358)	0.832 (0.630)	0.059 (0.023)	7.370 (15.351)	*** (***)
	RSLQ	→	Natural reward (RSLQ)	1.072 (0.930)	0.944 (0.895)	0.100 (0.032)	10.675 (28.711)	*** (***)
	RSLQ	→	Self-talk (RSLQ)	0.735 (0.594)	0.914 (0.769)	0.078 (0.028)	9.483 (21.019)	*** (***)
	RSLQ	→	Evaluation beliefs and assumptions (RSLQ)	0.897 (0.840)	0.957 (0.934)	0.081 (0.026)	11.091 (32.043)	*** (***)
	RSLQ	→	Visualizing successful performance (RSLQ)	1.106 (0.932)	0.882 (0.836)	0.130 (0.038)	8.504 (24.647)	*** (***)
	RSLQ	→	Self-punishment (RSLQ)	0.682 (0.577)	0.781 (0.696)	0.107 (0.033)	6.376 (17.698)	*** (***)
	PRCPS	→	Q9 (PRCPS)	1.000 (1.000)	0.725 (0.669)			
	PRCPS	→	Q8 (PRCPS)	1.284 (0.972)	0.720 (0.626)	0.315 (0.082)	4.069 (11.901)	*** (***)
	PRCPS	→	Q7 (PRCPS)	1.249 (0.839)	0.703 (0.601)	0.337 (0.075)	3.710 (11.119)	*** (***)
	PRCPS	→	Q6 (PRCPS)	1.362 (1.735)	0.622 (0.826)	0.426 (0.123)	3.195 (14.054)	0.001 (***)
	PRCPS	→	Q5 (PRCPS)	1.985 (1.446)	0.705 (0.603)	0.536 (0.133)	3.702 (10.893)	*** (***)
	PRCPS	→	Q4 (PRCPS)	0.439 (0.998)	0.244 (0.487)	0.343 (0.110)	1.280 (9.071)	0.201 (***)
	PRCPS	→	Q3 (PRCPS)	1.547 (1.520)	0.664 (0.694)	0.458 (0.122)	3.377 (12.454)	*** (***)
	PRCPS	→	Q2 (PRCPS)	1.735 (1.233)	0.664 (0.525)	0.486 (0.125)	3.568 (9.864)	*** (***)
	PRCPS	→	Q1 (PRCPS)	0.990 (1.283)	0.564 (0.709)	0.345 (0.103)	2.869 (12.495)	0.004 (***)

In both 2021 and 2022, male self-leadership had no significant influence on epidemic risk perception (Tables 7, 8, *p* = 0.377 and *p* = 0.450, respectively). In 2021, self-leadership of medical practitioners, people with an associate degree or below, or those with a master’s degree or above had no significant influence on epidemic risk perception (Tables 9, 11, *p* = 0.209, *p* = 0.354, and *p* = 0.193, respectively). In 2022, female epidemic risk perception had no significant effect on the quality of life (Table 8, *p* = 0.135), epidemic risk perception of people with an associate degree or below or a master’s degree or above had no significant effect on the quality of life (Table 10, *p* = 0.139 and *p* = 0.702, respectively), self-leadership of non-medical practitioners had no significant effect on epidemic risk perception (Table 12, *p* = 0.09), and epidemic risk perception of medical practitioners had no significant effect on quality of life (Table 12, *p* = 0.098).

### fsQCA of the relationship between nine dimensions of self-leadership and quality of life levels

Considering that a score of 2 means rarely experience, 3 means sometimes experience, and 4 means usually experience for each item of RSLQ, full non-membership, cross-over point, and full membership were set to 2.00, 3.00, and 4.00, respectively. In 2021, the average total score of quality of life was 62.62 ± 14.50 points; hence, full non-membership, cross-over point, and full membership were set to 32.62, 62.62, and 91.62, respectively, and the minimum effective configuration number was set to more than 30. In 2022, the average total score of quality of life was 60.30 ± 14.16 points, and full non-membership, cross-over point, and full membership were set to 31.98, 60.30, and 88.62, respectively, and the minimum effective configuration number was set to more than 4. The impact of self-leadership configuration on quality of life in 2021 and 2022 is detailed in Table 13. Through the analysis of survey data, a relatively consistent conclusion can be reached:

(1) The core condition of achieving high quality of life is that either self-punishment or self-cueing is at a high level and the other is at a low level. The marginal condition is that the seven dimensions of self-leadership (self-goal setting, self-observation, self-reward, natural reward, self-talk, evaluation beliefs and assumptions, and visualizing successful performance) exist simultaneously at a high level. The overall consistency of the two conditions was 87.10 and 87.03%, respectively, and the overall coverage was 39.95 and 41.27%, respectively.
(2) The core condition leading to set of non high QoL was the non high level of self-punishment or self-cueing, while the marginal condition was the non high level or high level of the other dimensions of self-leadership in the same path(~ High QoL: paths1-3); the overall consistency was 78.38% and 78.78%, and the overall coverage was 46.02% and 45.46%, respectively.

**Table 13:** 2021/2022 population self-leadership configurations affecting QoL.

Nine dimensions of self-leadership	High QoL		~High QoL		
Solution	1	2	1	2	3
Self-goal setting	•	•	⨂	•	•
Self-observation	•	•	⨂	•	•
Self-reward	•	•	⨂	•	•
Self-punishment	•	⨂	⨂	•	⨂
Self-cueing	⨂	•	⨂	⨂	•
Natural reward	•	•	⨂	•	•
Self-talk	•	•	⨂	•	•
Evaluation beliefs and assumptions	•	•	⨂	•	•
visualizing successful performance	•	•	⨂	•	•
Raw coverage	0.2973/0.3026	0.2721/0.3009	0.2161/0.2075	0.2895/0.2854	0.2419/0.2695
Unique coverage	0.1274/0.1118	0.1022/0.1101	0.1029/0.0880	0.1099/0.0928	0.0672/0.0804
Consistency	0.8546/0.8634	0.9200/0.9047	0.8833/0.8900	0.8334/0.8289	0.8194/0.8250
Overall solution coverage	0.3995/0.4127		0.4602/0.4546		
Overall solution consistency	0.8710/0.8703		0.7838/0.7878		

~High QoL indicates set of non high QoL. According to the expression of the QCA results suggested by Fiss^[25]^, black circles (“•”) indicate the presence of a condition, and circles with a cross-out (“⨂”) indicate its absence. Furthermore, large circles indicate core conditions, and small circles refer to peripheral conditions.

## Discussion

In this study, a mechanism was constructed to investigate the impact of self-leadership on pandemic risk perception and promotion of quality of life in public health emergencies, and the adaptation and stability of this mechanism among different populations over the past 2 years were explored. The higher epidemic risk perception and lower quality of life of people during large-scale COVID-19 transmission in 2022 are reasonable with main disease physical influence in China. The level of self-leadership of the public remained relatively stable during the study which could indentify changes in quality of life and epidemic risk perception are, to some extent, difficult to backfire on self-leadership.

From the detail results, it can be seen that the direct positive effect of self-leadership on quality of life is significant over the past 2 years, but the indirect effect of self-leadership on quality of life through pandemic risk perception is not satisfactory. Especially for men, the role of self-leadership in perceiving the risk of the pandemic is not significant. This may be related to the roles played by men in the family and social work; that is, excluding self-leadership factors, men can better cope with risk perception than women, and it could be due to differences between men and women in their fear of potential or immediate risks and in their perception of safety (26). Self-leadership is needed to directly regulate risk perception and one’s quality of life (both pathways have high significance). At the end of 2022 (during the peak of the COVID-19 outbreak in mainland of China), it was found that the epidemic risk perception among non-medical personnel significantly negatively impacted their quality of life, whereas the impact of risk perception on the quality of life of medical professionals was not significant (see Table 12). For those not related to the medical field, it is necessary to reduce their risk perception and adapt to the changes during an epidemic rationally and without stress to enhance their quality of life. This phenomenon may be attributed to the rapid spread of the virus among the Chinese population during the outbreak period, leading to heightened psychological burden and decreased quality of life regardless of infection status. As the number of patients seeking medical care sharply increased, medical professionals experienced both an increase in medical performance and heightened work pressure. Due to the nature of their work, medical professionals quickly adapt to this high-pressure environment, gradually mitigating the negative impact of epidemic risk perception on their quality of life (27). Additionally, prior widespread vaccination coverage among the Chinese population reduces the severity of disease symptoms after infection, thereby significantly alleviating the psychological burden of work.

The study by Hyesun Kang and colleagues shows that people with higher educational qualifications generally have higher levels of self-leadership (28); however, when considering the impact of self-leadership on quality of life, the difference is not significant. This indicates that self-leadership actions for managing and promoting personal health are not affected by educational level. Self-punishment and self-cueing are key dimensions of self-leadership. Self-cueing, as a positive factor, promotes the achievement of goals by strengthening one’s positive actions, which is a prominent condition for improving the quality of life. However, some studies (29, 30) have shown that self-punishment is negative and unfavorable in several fields such as health care and coping with stress. The study indicates that this does not entirely apply to health promotion and improvement of quality of life. Self-punishment is the process of reflecting on one’s aversion to insufficient performance in order to further summarize experiences and stimulate one’s motivation to achieve goals. This kind of thinking may not always be unfavorable, but can also be positive, and it is not applicable to all groups of people. In other words, only a portion of people are able to use this “negative motivation” to guide themselves toward better work and quality of life.

## Conclusion

Different from previous studies on the role of self-leadership in improving work efficiency and action motivation (31–37), this study innovatively applies the self-leadership in improving people’s life quality in the post-epidemic era, and the qualitative comparative analysis based on cases more truly positions the prominent effects of self-punishment and self-cueing and explains the limitations of self-punishment. Under the public health policy, the improvement of people’s quality of life can be started from the popularization of self-leadership education and publicity, which is in line with the current proactive health concept of “being the first person for one’s own health” (38) and will bring overall social and economic benefits and reduce the burdens of social environment management.

This study has several limitations. First, this was a cross-sectional study and the causal relationship among the variables could not be determined. Moreover, there may be other factors that influence self-leadership, risk perception, and quality of life. For example, people with different quality of life levels may have differences in their self-leadership and pandemic risk perception. Second, due to the epidemic control measures at the time of the surveys, face-to-face investigation was not possible. Therefore, the data of this study were mainly collected through online questionnaires with inevitable bias of subjective recall. The influence of self-leadership on quality of life should be further explored in the future “non-pandemic period.” Third, snowball and convenient sampling methods were used to recruit participants that caused lack of population-wide representation partly. Therefore, the generalization of results warrants caution. In addition, the cognitive development and judgment ability of people of different ages are easy to be limited by educational level and regional culture, which are also potential factors affecting the stability of the results. In future research, random sampling can be used to explore the ways various people cope with stressors and promote the improvement of life of quality by formulating and implementing self-leadership intervention plans.

In the post-pandemic era, improving people’s self-leadership is generally beneficial for improvement in their quality of life. In the future, further studies are needed to explore the population characteristics of those who use self-punishment strategy to achieve good health outcomes, so as to accurately utilize self-leadership strategy to achieve health management goals.

## Data availability statement

The original contributions presented in the study are included in the article/Supplementary material, further inquiries can be directed to the corresponding author.

## Ethics statement

The studies involving humans were approved by Medical Ethics Committee of Kaifeng Hospital of Traditional Chinese Medicine. The studies were conducted in accordance with the local legislation and institutional requirements. The participants provided their written informed consent to participate in this study.

## Author contributions

JR: Conceptualization, Data curation, Formal analysis, Investigation, Methodology, Validation, Visualization, Writing – original draft, Writing – review & editing. YZ: Conceptualization, Funding acquisition, Investigation, Project administration, Supervision, Writing – original draft. YH: Investigation, Writing – review & editing. XZ: Investigation, Writing – review & editing. GP: Investigation, Writing – review & editing. LL: Investigation, Writing – review & editing. QZ: Investigation, Writing – review & editing.

## Footnotes

^1^www.wjx.cn
